# Analysis of the clinical characteristics of pembrolizumab-induced bullous pemphigoid

**DOI:** 10.3389/fonc.2023.1095694

**Published:** 2023-03-03

**Authors:** Jianglin Wang, Xin Hu, Wei Jiang, Wenjie Zhou, Mengjie Tang, Cuifang Wu, Wei Liu, Xiaocong Zuo

**Affiliations:** ^1^ Department of Pharmacy, The Third Xiangya Hospital, Central South University, Changsha, China; ^2^ Department of Pharmacy, Taojiang County People’s Hospital, Yiyang, China; ^3^ Department of Pharmacy, Nanxian Hospital of Traditional Chinese Medicine, Yiyang, China; ^4^ Department of Pharmacy, Yongzhou Third People’s Hospital, Yongzhou, China; ^5^ Department of Pathology, Hunan Cancer Hospital, The Affiliated Cancer Hospital of Xiangya School of Medicine, Central South University, Changsha, China

**Keywords:** pembrolizumab, bullous pemphigoid, clinical characteristics, treatment, pathogenesis

## Abstract

**Background:**

Pembrolizumab, a programmed cell death protein 1 checkpoint inhibitor, is a novel drug used to treat a variety of advanced malignancies. However, it can also result in many immune-related adverse events, with cutaneous toxicities being the most frequent. Regarding pembrolizumab-induced skin adverse reactions, bullous pemphigoid (BP) has the worst effects on quality of life. Recently, there have been more and more reports of BP incidents resulting from pembrolizumab therapy in patients with cancer. This study aimed to define the clinical characteristics, diagnosis and management of pembrolizumab-induced BP and identify potential differences between classical BP and pembrolizumab-induced BP.

**Methods:**

Case reports, case series, and case analyses of pembrolizumab-induced BP up to 10 December 2022 were collected for retrospective analysis.

**Results:**

Our study included 47 patients (33 males and 14 females) from 40 studies. The median age was 72 years (range 42-86 years). The median time to cutaneous toxicity was 4 months (range 0.7-28 months), and the median time to bullae formation was 7.35 months (range 0.7-32 months). The most common clinical features were tense bullae and blisters (85.11%), pruritus (72.34%), and erythema (63.83%) on the limbs and trunk. In 20 of the 22 cases tested, the serum anti-BP180 autoantibodies were positive. However, in 10 cases (91.90%, 10/11) the circulating autoantibodies of anti-BP230 were negative. 40 patients had skin biopsies and the skin biopsy revealed subepidermal bullae or blister eosinophil infiltration in 75.00% of patients with pembrolizumab-induced BP, 10.00% of patients with lymphocyte infiltration and 20.00% of patients with neutrophil infiltration. There were 20 patients (50%) with eosinophilic infiltration around the superficial dermis vessels, 8 patients (20.00%) with lymphocyte infiltration around the superficial dermis vessels, and 4 patients (10.00%) with neutrophil infiltration around the superficial dermis vessels. Direct immunofluorescence detected linear immunoglobulin G (IgG) IgG and/or complement C3 along the dermo-epidermal junction in 36 patients (94.74%) with BP. IgG positivity was detected by indirect immunofluorescence in 81.82% of patients with BP. All patients were in complete remission (95.65%,44/46) or partial remission (4.35%, 2/46) of BP, whereas 9/46 patients had a relapse or refractory. The majority of patients achieved BP remission after discontinuation of pembrolizumab with a combination of topically and systemically administered steroid treatments, or other medications. The median duration of BP remission was 2 months (range 0.3-15 months).

**Conclusion:**

A thorough diagnosis of pembrolizumab-induced BP should be made using clinical signs, biochemical markers, histopathological and immunopathological tests. Pembrolizumab-induced BP had similar clinical characteristics to classic BP. Temporary or permanent discontinuation of pembrolizumab therapy may be required in patients with perbolizumab-induced BP depending on the severity of BP and the response to medication. Pembrolizumab-induced BP may be effectively treated using topical and systemic steroid treatments in combination with other medications (e.g., doxycycline, niacinamide, dapsone, rituximab, intravenous immunoglobulins, dupilumab, cyclophosphamide, methotrexate, mycophenolate mofetil, and infliximab). Clinicians should provide better management to patients with BP receiving pembrolizumab to prevent progression and ensure continuous cancer treatment.

## 1 Introduction

The most common autoimmune subepidermal bullous disease affecting the skin and mucous membranes are bullous pemphigoid (BP). Autoantibodies that target the BP180 and/or BP230 at the dermal-epidermal junction induce BP (1). Clinically, patients with BP often present with tense blister formation and symptoms of severe pruritus over urticarial plaques on the trunk and extremities (2). According to by Lu et al., the overall incidence was 4.19 per 100 000 person-years (3). Additionally, the prevalence of BP rises dramatically with aging and is most common among the elderly (4–6). Although the etiology of BP has not been completely clarified, recent studies and case reports have suggested that neurologic disorders; radiation therapy; burns; ultraviolet exposure; infections; trauma; surgical procedures; and medications like dipeptidyl peptidase-4 inhibitors, non-steroidal anti-inflammatory drugs, diuretics (e.g., furosemide), angiotensin receptor blocker, angiotensin-converting enzyme inhibitors, antibacterial agents (e.g., ciprofloxacin and amoxicillin), potassium iodide, D-penicillamine, and biological therapy like anti-tumor necrosis factor drugs, etc. are known to trigger the development of BP (7, 8)

Pembrolizumab, a highly specific, humanized monoclonal immunoglobulin G4κisotype antibody checkpoint inhibitor, prevents interaction of the programmed death 1 (PD-1) receptor with the programmed death-ligand 1 (PD-L1) and PD-L2 ligands enabling T-cell-mediated immune response against tumor cells (9, 10). In treating various cancer types or late-stage and metastatic cancers, it has been frequently prescribed as a vital component of the standard of care (11). Although pembrolizumab has a better overall survival rate, lower toxicity, and a higher quality of life when compared to chemotherapy (12), this agent has a novel specific spectrum of side effects called immune-related adverse events (irAEs), which are caused by the nonspecific activation of the immune system (13, 14). Immune checkpoint inhibitors (ICIs)-treated patients are more likely to experience cutaneous toxicity than other irAEs, with a 50% incidence rate (14). Although several cases analyzing the association between pembrolizumab therapy and BP have been reported recently, pembrolizumab-induced BP is still easily overlooked in clinical practice, delaying diagnosis and treatment. It is important to improve understanding and management of pembrolizumab-induced BP since this rare skin toxicity may significantly impact on future therapeutic management and oncological prognosis. This study aimed to investigate the clinical characteristics, diagnosis, pathogenesis, and management of pembrolizumab-induced BP. Furthermore, we will attempt to identify any possible differences between classical BP and pembrolizumab-induced BP.

## 2 Methods

### 2.1 Search strategy

We searched PubMed/MEDLINE, Web of Knowledge, OVID, Elsevier, Springer Link, EMBASE, Cochrane Library, China National Knowledge Infrastructure (CNKI), Wanfang Data, and Chinese VIP databases in Chinese and English from inception to 10 December 2022. Bullous pemphigoid, pembrolizumab, PD-1 inhibitor, and PD-L1 inhibitor were some of the subject words and free words we combined to find the literature on BP induced by pembrolizumab. The search involved all fields, including title, abstract, keywords, and full text.

### 2.2 Inclusion and exclusion criteria

The preliminary study included case reports, case series, and case analyses of pembrolizumab-induced BP. Duplicate literature, reviews, observational studies, mechanistic studies, animal studies, and full-text articles with insufficient data were excluded. Articles written in languages other than English and Chinese were also excluded.

### 2.3 Data extraction

Two investigators extracted data independently using a self-designed data extraction table. A panel discussion resolved investigator disagreements. From each paper: country, age, gender, medical history, primary disease, pembrolizumab dosage, onset time, clinical manifestations, laboratory examination, skin biopsy, treatment, and prognosis were extracted.

### 2.4 Statistical analysis

The Statistical Package for Social Sciences (version 22.0 IBM Corp: Armonk, NY, USA) was used to analyze the acquired data statistically. The results of quantitative variables were expressed as median values and range (minimum and maximum), while categorical variables were represented using the number of cases and percentages.

## 3 Results

### 3.1 Clinical information

Ultimately, 40 studies with 39 case reports and 1 case series met the eligibility criteria (2, 15–53). Totally, 47 patients (33 males and 14 females) were enrolled in the study (**Table 1**). The median age of the cases was 72 years (range 42-86 years), and cases older than 60 accounted for 87.23% of the study population. The cases originated in the following locations: 16 from North America, 13 from Europe, 10 from Asia, 6 from Oceania, and 2 from South America. According to the indicator for pembrolizumab, 25 patients received treatment for melanoma, 12 received treatment for lung carcinoma, 4 received treatment for bladder urothelial cancer, 2 received treatment for renal-cell carcinoma, 1 received treatment for endometrial cancer, 1 received treatment for cervical cancer, 1 received treatment for intrahepatic cholangiocarcinoma, and 1 was unavailable. The regimen and dosage of pembrolizumab were chosen following product recommendations or authoritative guidelines. The onset of cutaneous toxicity symptoms varied significantly from 0.7 months to 28 months after starting pembrolizumab therapy. The median onset time of cutaneous toxicity was 4 months. It is important to note that the median time for bullae formation was 7.35 months (range 0.7-32 months). Approximately 72.09% of patients had their pembrolizumab discontinued when they were diagnosed with BP, and the interval between BP diagnosis and pembrolizumab termination was 0-11 months. Interestingly, although 82.98% of BP cases developed or flared while receiving pembrolizumab treatment, 8 patients (17.02%) experienced the onset of BP after discontinuing the drug. The median time for BP after pembrolizumab discontinuation was 1.7 months (0.7-5 months). Comorbidities were reported in 10 cases. The comorbidities included skin diseases (40.00%), hypertension (30.00%), type 2 diabetes (30.00%), chronic renal insufficiency (20.00%), hypothyroidism (10.00%), Crohn’s disease (10.00%), status epilepticus (10.00%). Simultaneously, 3 of these 10 patients took medication that may cause BP, including teneligliptin, candesartan, enalapril, aspirin, nicorandil and cephalexin. Additionally, 4 patients took an unidentified drug to treat hypertension, type 2 diabetes, and status epilepticus.

**Table 1:** Characteristics of the 47 reported in case series/reports.

Parameter		Value
Gender	Male	33 (70.21%)
	Female	14 (29.79%)
Region	North America	16 (34.04%)
	Europe	13 (27.66%)
	Asia	10 (21.28%)
	Oceania	6 (12.77%)
	South America	2 (4.26%)
Age (years)	Median age	72 (42,86)^a^
	≤60	6 (12.77%)
	61-70	14 (29.79%)
	71-80	20 (42.55%)
	≥81	7 (14.89%)
Type of cancer	Melanoma	25 (53.19%)
	Lung carcinoma	12 (25.53%)
	Bladder urothelial cancer	4 (8.51%)
	Renal-cell carcinoma	2 (4.26%)
	Endometrial cancer	1 (2.13%)
	Cervical cancer	1 (2.13%)
	Intrahepatic cholangiocarcinoma	1 (2.13%)
	NA	1 (2.13%)
Time to cutaneous toxicity (40)^b^	Median time (months)	4(0.7,28)^a^
	≤2	13 (31.71%)
	3-7	12 (29.27%)
	8-11	6 (14.63%)
	14-18	6 (14.63%)
	≥20	4 (9.76%)
Time to bullae (45)^b^	Median time (months)	7.35 (0.7,32)^a^
	≤3	8 (17.78%)
	3-6	12 (26.67%)
	6-12	12 (26.67%)
	14-18	6 (13.33%)
	≥20	7 (15.56%)
Time from BP diagnosis to pembrolizumab termination	Median time (months)	0 (0,11)^a^
Whether to discontinue pembrolizumab or not	Yes	39 (82.98%)
	Not	4 (8.51%)
	NA	4 (8.51%)
Comorbidities (10)^b^	Hypertension	3 (30.00%)
	Chronic renal insufficiency	2 (20.00%)
	Skin diseases	4 (40.00%)
	Type 2 diabetes	3 (30.00%)
	Crohn’s disease	1 (10.00%)
	Hypothyroidism	1 (10.00%)
Neurologic diseases	1 (10.00%)	

BP, bullous pemphigoid; NA, not available.

a Median (minimum, maximum).

b Indicates the number of 47 patients for whom information on the particular parameter was provided.

### 3.2 Clinical presentation and laboratory tests

This study included 47 patients whose clinical presentations and the laboratory tests results are described in **Table 2**. Patients with BP most frequently had tense bullae and blisters (85.11%), pruritus (72.34%), and erythema (63.83%). These patients had a skin erosion or rupture (57.45%), oral mucosal (19.15%), and eczema (8.51%) in certain cases. Obvious pain was present in 5 patients. Skin lesions could spread throughout the body, including the limbs, trunk, back, chest, etc., could all develop skin lesions. Skin lesions mainly developed in the limbs (78.26%) and trunk (47.83%) of our 46 patients. The laboratory test results showed that 20 patients (90.91%) with BP tested positive for anti-BP180 autoantibodies (**Table 2**). Among these, only one tested positive for BP180NC16a, a key BP antigen that is a dominant antigenic determinant in BP serum. However, in 10 patients (91.90%) with pembrolizumab-induced BP, anti-BP230 autoantibodies detection was negative.

**Table 2:** Clinical information on the 47 included patients.

Parameter	Clinical features	Value
Clinical presentation (47)^a^	Tense bullae and blisters	40(85.11%)
	Pruritus	34(72.34%)
	Erythema	30(63.83%)
	Skin erosion or rupture	27(57.45%)
	Oral mucosal lesions	9(19.15%)
	Pain	5(10.64%)
	Eczema	4(8.51%)
Skin lesion distribution (46)^a^	Whole body	7(15.22%)
	Limbs	36(78.26%)
	Trunk	22(47.83%)
	Back	13(28.26%)
	Chest	11(23.91%)
	Abdomen	5(10.87%)
	Face/neck/head/shoulder	12(26.09%)
Laboratory testing	BP180 or BP180NC16a positive (22)^a^	20(90.91%)
	BP230 positive (11)^a^	1(9.10%)
	Anti-desmoglein 1 and 3 antibodies positive (4)^a^	0(0%)
	Eosinophilia (6)^a^	2(33.33%)
	Leukocytosis (5)^a^	1/5(20%)
Skin biopsy		
Epidermis (40)^a^	Subepidermal blister	37(92.5%)
	Subepidermal blister with eosinophil infiltration	30(75.00%)
	Subepidermal blister with neutrophil infiltration	8(20.00%)
	Subepidermal blister with lymphocyte infiltration	4(10.00%)
	Subepidermal blister with fibrin	2(5.00%)
	Chronic inflammation with eosinophils	1(2.50%)
	Spongiosis	3(7.50%)
	Epidermal hyperplasia and edema	1(2.50%)
Dermis (40)^a^	Eosinophilic infiltration around the superficial dermis vessels	20(50.00%)
	Lymphocyte infiltration around the superficial dermis vessels	8(20.00%)
	Neutrophil infiltration around the superficial dermis vessels	4(10.00%)
	Mononuclear cells infiltration around the superficial dermis vessels	2(5.00%)
Immunofluorescence	DIF (38)^a^	
	IgG and C3 positive	26(68.42%)
	IgG, C3 and C4 positive	1(2.63%)
	IgG, C3 and IgA positive	2(5.63%)
	IgG, C3, IgA and IgM positive	1(2.63%)
	IgG and C3 negative	2(5.63%)
	IgG posive and C3 negative	1(2.63%)
	IgG negative and C3 positive	1(2.63%)
	Unspecific IgG and C3 positive	2(5.63%)
	IgG positive^b^	1(2.63%)
	C3 positive^b^	1(2.63%)
	IIF (11)^a^	
	IgG positive	7(63.64%)
	IgG and IgA positive	1(9.10%)
	IgG positive and IgA negative	1(9.10%)
	IgA positive^b^	1(9.10%)
	IgG negative	1(9.10%)
	Unspecific DIF and IIF (1)	
	IgG positive^b^	1(100.0%)
Immunohistochemical stain	C3d^10^ positive (1)	1(100.0%)

BP, bullous pemphigoid; DIF, direct immunofluorescence; IIF, indirect immunofluorescence.

a Indicates the number of 47 patients for whom information on the particular parameter was provided.

b Other information on DIF or IIF was not available.

### 3.3 Hist opathological analysis and immunofluorescence

The result of histopathological analysis and immunofluorescence was shown in **Table 2**. Skin biopsy was performed on 40 patients (**Table 2**). Among them, 37 patients (92.50%) displayed subepidermal blisters, 30 patients (75.00%) displayed subepidermal blisters with eosinophil infiltration, 8 patients (20.00%) displayed subepidermal blisters with neutrophil infiltration, 4 patients (10.00%) displayed subepidermal blisters with lymphocyte infiltration, and 2 patients (5.00%) displayed subepidermal blisters with fibrin. Simultaneously, 1 patients (2.50%) displayed chronic inflammation with eosinophils, 3 patients (7.50%) displayed spongiotic dermatitis with a mixed-cell infiltrate, and 1 patients (2.50%) displayed epidermal hyperplasia and edema. Additionally, 20 patients (50.00%) had eosinophilic infiltration around the superficial dermis vessels, 8 patients (20.00%) had neutrophil infiltration around the superficial dermis vessels, 4 patients (10.00%) had lymphocyte infiltration around the superficial dermis vessels, and 2 patients (5.00%) had mononuclear cells infiltration around the superficial dermis vessels.

Direct immunofluorescence (DIF) was performed on 38 patients. Of these, 29 patients (78.38%) in the punch biopsy of skin exhibited both linear immunoglobulin G (IgG) and complement C3 (C3) deposits at the subepidermal basement membrane zone, and one patient also had C4 deposits, 2 also had IgA deposits, and one also had IgA and IgM deposits. Only one case had linear IgG, and only one had linear C3 deposition, respectively. However, 2 patients exhibited DIF negative. IgG results of one patient and C3 results of another patient was positive. Moreover, 2 patients had DIF positive with unspecific IgG and C3. Indirect immunofluorescence (IIF) was performed on a total of 11 patients. Of these, 9 patients exhibited IgG positive, including one patient who also had IgA positive, and one patient who had IgA negative. One of these patients had an IIF-negative presentation. Only a single patient’s positive IgA data was available to us. Only one publication reported the immunofluorescence (IF) results and showed IgG positive. An immunohistochemistry stain test for C3d10 was conducted in only 1 patients and the results demonstrated robust linear staining along the dermal-epidermal junction.

### 3.4 Treatment

**Table 3** lists the treatment and prognosis of the 47 patients. When BP was identified in 20 patients (46.51%), pembrolizumab was discontinued, and in 4 patients (9.30%), the drug was temporarily discontinued, then reinitiated. Seven patients (16.28%) attempted to continue pembrolizumab therapy while still received symptomatic treatment simultaneously. However, they ultimately failed (**Table 3**). Continual pembrolizumab treatment was administered to only 4 patients (9.30%). Forty-three patients (91.49%) received systemic therapy, 42 (89.36%) received systemic corticosteroids, of which 20 patients had slow tapering-off regimens, and 4 patients experienced poor efficacy after receiving corticosteroid therapy. Tetracyclines (12, 25.53%), niacinamide (5, 10.64%), dapsone (2, 4.26%), mycophenolate mofetil (2, 4.26%), cyclophosphamide (1, 2.13%), methotrexate (1, 2.13%), hydroxyzine(1, 2.13%), and immune globulin intravenous (1, 2.13%) were given to 21 patients as nonbiologic treatments. Furthermore, 5 patients (10.64%) with severe or refractory received biological therapy, including rituximab (3, 6.38%), dupilumab (1, 2.13%), and infliximab (1, 2.13%). Topical steroid therapy was administered to 35 patients (74.47%). Fifteen patients who were reported had BP remission at a median time of 2 months (range 0.3-15 months).

**Table 3:** Treatment and prognosis of the 47 reported in case series/reports.

Parameter		Value
Pembrolizumab management when BP diagnosed(43)^a^	Discontinued immediately	20(46.51%)
	Discontinued after a period of time for continuous treatment by pembrolizumab	7(16.28%)
	Continued treatment	4(9.30%)
	Temporarily discontinued then reinitiated	4(9.30%)
	Onset after the cessation of pembrolizumab	8(18.60%)
Treatment(47)^a^	**Topical steroid treatment**	35(74.47%)
	**Systemic treatment**	43(91.49%)
	Systemic corticosteroid treatment	42(89.36%)
	Nonbiologic treatments	21(44.68%)
	Tetracyclines	12(25.53%)
	Niacinamide	5(10.64%)
	Dapsone	2(4.26%)
	Mycophenolate mofetil	2(4.26%)
	Cyclophosphamide	1(2.13%)
	Methotrexate	1(2.13%)
	Hydroxyzine	1(2.13%)
	Immunoglobulin	1(2.13%)
	Biologic treatments	5(10.64%)
	Rituximab	3(6.38%)
	Dupilumab	1(2.13%)
	Infliximab	1(2.13%)
BP remission time	Median time (months)	2(0.3,15)b
BP outcome(47)^a^	**Partial to complete remission**	37(78.72%)
	Discontinued immediately	16(34.04%)
	Discontinued after a period of time for continuous treatment by pembrolizumab	6(12.77%)
	Continued treatment	4(8.51%)
	Temporarily discontinued then reinitiated	3(6.38%)
	Onset after the cessation of pembrolizumab	5(10.64%)
	No information was available on whether or not to discontinue the pembrolizumab	3(6.38%)
	**Relapse/refractory symptoms^c^**	9(19.15%)
	Discontinued immediately	3(6.38%)
	Discontinued after a period of time for continuous treatment by pembrolizumab	1(2.13%)
	Temporarily discontinued then reinitiated	1(2.13%)
	Onset after the cessation of pembrolizumab	3(6.38%)
	No information was available on whether or not to discontinue the pembrolizumab	1(2.13%)
	**Not report**	
	Discontinued immediately	1(2.13%)
Treatment response of pembrolizumab (27)^a^	Complete response, partial response or stable disease	17(62.96%)
	Progressive disease	9(33.33%)
	No response	1(3.70%)

BP, bullous pemphigoid.

^a^Indicates the number of 47 patients for whom information on the treatment and prognosis was provided.

^b^Median (minimum, maximum).

Includes patients who had appeared flares of BP on attempted tapering of systemic glucocorticoids.

Sixteen patients had partial (1 patient) or complete (15 patients) improvement in BP, 1 patient did not report a BP prognosis, and 3 patients had relapsed (2 patients)/refractory (1 patient) BP among the patients who had pembrolizumab induced-BP and immediately discontinued pembrolizumab. Seven patients who experienced pembrolizumab induced BP attempted to continue taking pembrolizumab while receiving systemic glucocorticoids therapy, but they all discontinued due to recurrent BP. Following the withdrawal of pembrolizumab, 6 patients experienced partial or complete remission of BP, while 1 patient continued to experience relapses despite attempts to taper off their systemic glucocorticoids. Four patients who continued taking pembrolizumab after developing pembrolizumab-associated BP showed complete improvement in BP with topical and/or systemic glucocorticoids treatment. When pembrolizumab use was resumed, BP recurred in 4 patients. Of the 8 patients who developed BP following the withdrawal of pembrolizumab, 5 experienced partial (1 patient) or complete (4 patients) remission and 3 patients experienced relapsed (1 patient)/refractory (2 patients) BP. Moreover, 3 patients had a complete response, and one had a relapsed BP after an attempt to taper off systemic glucocorticoids among the 4 patients who had no information on whether to discontinue using pembrolizumab. The response to pembrolizumab-based cancer treatment was reported in 27 patients. Seventeen (62.96%) of them had complete responses, partial responses or stable diseases (SD), 9 (33.33%) had progressive diseases (PD) and 1 patient (3.70%) had no response.

## 4 Discussion

Retrospective studies indicate that although BP is a rare irAE with a prevalence of approximately 0.6%, it is a well-established irAE associated with PD-1 and PD-L1 inhibition (54, 55). The present study, which included 47 patients, demonstrated pembrolizumab therapy could cause BP. Interestingly, studies have linked irAEs to higher rates of treatment success and improved survival following pembrolizumab therapy (56, 57). Furthermore, it has been suggested that the occurrence of irCAEs may be a useful indicator for predicting improved tumor response and better survival among cancer patients receiving ICIs immunotherapy (57, 58). However, the effectiveness of irAEs as a predictor was reduced because irAEs occurred only after ICIs treatment commencement. Additionally, a cohort and nested propensity score-matched case-control study have demonstrated a significant association between tumor response to ICIs and the development of ICIs-induced BP in any phase of the patient’s treatment (54). Our findings showed that only 62.96% of patients with pembrolizumab-induced BP would exhibit a complete/partial response or stable condition after pembrolizumab treatment, indicating that not all patients with BP induced by pembrolizumab would respond favorably to pembrolizumab. Therefore, additional research is required to determine whether pembrolizumab-induced BP was associated with the therapeutic response to pembrolizumab.

The majority of cases of classic BP have been documented in females, with a female-to-male ratio ranging between 1.04 and 5.1 (55). However, our analysis shows that males are more likely to develop pembrolizumab-induced BP. Several factors could contribute to this phenomenon. First, the results of this study can be biased because of the small sample size. Additionally, pembrolizumab-induced BP is primarily found in patients with melanoma and lung carcinoma, with a significant male predominance (59, 60). Therefore, this is a significant factor in the skewed sex distribution towards males caused by pembrolizumab-induced BP. However, Duma et al. performed a retrospective review to examine sex differences in irAEs in all patients with metastatic melanoma, or non-small-cell lung cancer treated with PD-1 and PD-L1 inhibitors, and they discovered that females had a higher risk of irAEs than males did for both the conditions (61). According to the literature, there is a debate regarding whether there are sex differences in the irAEs of ICI therapy. Further research is required to determine whether there is sex-related differences in pembrolizumab-induced BP.

Age is frequently seen as a risk factor for BP (54). According to several previous studies, the prevalence of BP rises progressively and considerably with age in the general population, notably in people over 70 (3, 62–64). According to Lu et al., age-specific incidence increased across all age groups. The incidence of BP was lowest in those under 50 but increased significantly after age 70 (3). However, the mechanism behind the association between age and BP remains unclear. One hypothesis is that aging profoundly affects the integrity and function of the skin and is accompanied by immunosenescence, a dysregulation of the immune system that may increase the incidence of BP in the elderly (65). Another hypothesis is that older people have a higher risk of diseases, including neurological and cardiovascular disorders, which may be connected to BP (63). Our findings demonstrated that pembrolizumab-induced BP mostly impacted elderly patients approximately 72 years old, like classical BP. Previous studies have also shown that those who take ICIs and are 70 years or older have an increased chance of developing idiopathic BP (54, 66). It implies that the underlying tendency of BP development in the elderly may exacerbate by ICIs.

Loss of immunological tolerance is associated with the pathogenesis of BP and may result in the development of pathogenic IgG1 and IgG4 autoantibodies directed against 2 autoantigens at the basement membrane zone (BMZ). The 2 autoantigens previously mentioned above are BP antigen 180 (BP180, also known as BPAG2 or type XVII collagen) and BP antigen 230 (BP230, also known as an epithelial isoform of BPAG1, BPAG1e), which are parts of the junctional adhesion complexes known as hemidesmosome proteins promoting dermo-epidermal junction. The binding of BP180 and BP230 to autoantibodies sets off a series of immunological processes that eventually encourage the breakdown of BMZ, leading to blister formation (65). Although using of pembrolizumab is a significant risk factor for the development of BP, the molecular markers and pathomechanisms underlying the association between pembrolizumab and BP have not yet been identified (67). One possible reason was that it was probably secondary to unintended consequences of B- and T-cell activity in an immunologically stimulated environment (68). Pembrolizumab increases B-cell receptor responses, initiates B-cell growth, and triggers the production of antibodies that cause cross-reactive immunogenicity against the basal membrane of the skin (69). Alternatively, pembrolizumab has been shown to have a negative impact on the ability of follicular helper T-B cells to select potentially mutated B cells and their ability to inhibit follicular regulatory T-B cells’ ability to do so. It promotes abnormal low-affinity plasma cells production, activating BP-mediated antibodies like anti-BP180 and anti-BP230 (69).

The current study also revealed that melanoma and lung carcinoma were the most frequent underlying malignancy in patients with pembrolizumab-induced BP. It is consistent with earlier research on immunotherapy-associated bullous disorders (70). In addition, Geisler et al. showed that primary tumor types, such as melanoma, non-small cell lung carcinoma, urothelial carcinoma, and head and neck squamous cell carcinoma were associated with BP eruptions (71). It is currently unknown why some patients develop pembrolizumab-associated BP while others do not. It could be related to the target antigen found at the dermo-epidermal junction and on tumor cells. Some studies discovered that malignant melanocytic tumor cells, non-small cell lung cancer cells, and urothelial epithelium all expressed BP180 on the surface (72–75). According to the “same-antigen-theory”, when the immune response to the tumor is generated, the immunogenicity of BP180 expressed by some tumor cells is activated. This causes a cross-reaction and causes the immune system to attack the BMZ, which results in the development of BP (76). Another reason could be that these 2 types of tumors are the most common for which pembrolizumab is currently prescribed.

It is known that, except for aging, male, and specific tumor, there are many predisposing factors for the development of BP, including genetics, aging, and comorbidities. The onset of BP may be influenced by genetic susceptibility. In population studies, HLA class II alleles (i.e., HLA-DR, -DP, and -DQ) have been associated with BP in several ethnic groups in population studies. Particularly, the HLA class II allele HLA-DQB1*03:01 has a strong association with BP. While HLADRB1*04, DRB1*1101, and DQB1*0302 were found more frequently in Japanese patients with BP. This shows that various HLA genotypes have distinct impacts on BP susceptibility in various ethnic groups (77). Unfortunately, there was no information on genetic testing in our cases. Although this association has not yet undergone thorough investigation in patients with immunotherapy-induced BP, HLA typing may be a sign of increased genetic susceptibility in the general population. On the other hand, a significant population-based case-control study conducted by Langan and colleagues revealed that patients with dementia, Parkinson’s disease, stroke, and epilepsy had a higher risk of developing BP (78). Our investigation includes a case of a patient with BP who was treated with an unidentified antiepileptic drug while in status epilepticus. It is unknown if these concomitant diseases or medications increase the risk of pembrolizumab-induced BP. BP is frequently caused by the assumption of systemic therapies, including antibiotics, beta-blockers, non-steroidal anti-inflammatory drugs, diuretics and, more recently, anti-tumor necrosis factor-α, dipeptidyl peptidase 4 inhibitors, and ICIs (79). Approximately 21.28% of the patients in this study who had pembrolizumab-induced BP had trigger factors.

The clinical presentation of pembrolizumab-induced BP resembled mostly classical BP, including a prodromal, pruritic, papular or eczematous eruption followed by moderate-to-severe symptomatic blisters involving more than 10% body surface area, which lesions predominantly involved the trunk and/or extremities. However, only a few patients (19.15%) exhibited oral mucosal lesions, comparable to the 10% to 25% of patients who had classic BP involving the mucosa (7). Interestingly, we discovered a difference in the median interval between the administration of pembrolizumab and the beginning of pruritus and bullous lesions. Bullous lesions typically emerge within the first 7 months of pembrolizumab therapy; the median time is 7.35 months, with some patients lasting even more than 20 months. In general, these results support earlier findings (67, 70). In our study, 8 patients developed BP after completing their pembrolizumab treatment or shortly after it was discontinued, which is consistent with the delayed onset of ICIs-induced cutaneous toxicity that has been commonly reported. However, pruritus usually preceded or occurred concurrently with BP development, and the median time between initiating therapy with pembrolizumab and pruritus onset was 4 months. In addition, a smaller subset of patients with BP symptoms limited to pruritus and rash did not develop bullous following initiation of pembrolizumab therapy. It can be challenging to distinguish pruritus and nonspecific rash from other cutaneous toxicities. Notably, patients with intractable pruritus should consider non-bullous types of BP. A skin biopsy should be examined histologically to see if any relevant pathogenic autoantibodies were detected. Immunotherapy interruption might be decreased with early diagnosis and treatment. Therefore, a dermatologic evaluation for BP may be premeditated in patients with chronic or atypical pruritus.

The DIF assays that detect autoantibodies is highly sensitive in diagnosing BP (80). Our study revealed that 78.95% of patients with pembrolizumab-induced BP had positive linear deposition of both IgG and C3 at the dermo-epidermal junction using DIF. In pembrolizumab-induced BP, the histologic analysis of a skin biopsy revealed subepidermal bulla, blister or vesicles along with inflammatory cell infiltration that included eosinophils, lymphocytes, and neutrophils and dermal lesions with the same inflammatory cell infiltration. An important characteristic of classical BP is the presence of circulating anti-BP180 and/or anti-BP230 autoantibodies. The serological examination typically detects anti-BP180 autoantibodies (71%-83%) and occasionally anti-BP230 autoantibodies (29.0%) in patients with PD-1 inhibitor-induced BP (14, 66, 76). These results also demonstrated that the anti-BP180 antibody had a significantly higher positive rate (90.91%) than the anti-BP230 antibody positive rate (9.10%) in pembrolizumab-induced BP. These findings imply that anti-BP180 autoantibodies may have a higher detection value than anti-BP230 autoantibodies in pembrolizumab-induced BP.

The primary objectives are to stop the development of new lesions, control skin eruption and pruritus, reduce major serious side effects from the treatment, and improve patients’ quality of life. However, the best course of action for pembrolizumab-induced BP has not been established. Patients were usually managed according to the severity of BP as determined by the Common Terminology Criteria for Adverse Events(CTCAE) (81). Unfortunately, the severity of BP was not reported in most cases included in this study. Although our findings indicated that pembrolizumab-induced BP flares and worsening could occur after pembrolizumab discontinuation or continuation, ceasing pembrolizumab therapy is typically an option for pembrolizumab-induced BP development in most cases. The findings of this study also indicated that partial/complete remission was difficult to attain in 7 patients with BP who did not stop using the pembrolizumab immediately. These patients had a progressive remission of BP only if they discontinued taking pembrolizumab. Furthermore, our findings revealed that BP flares when pembrolizumab is administered to 4 patients again. Only a tiny percentage of patients (8.51%) who develop BP can continue taking pembrolizumab while receiving treatment with glucocorticoids. Despite this, the clinician still needs to determine whether to discontinue pembrolizumab permanently or only temporarily based on the patient’s tumor control and the severity of BP.

The treatment drugs for pembrolizumab-induced BP are basically similar to classical BP in that topical steroids and systemic corticosteroids are the most common first-line treatment, frequently in conjunction with other drugs (i.e., cyclophosphamide, methotrexate, azathioprine, mycophenolate mofetil, immune globulin intravenous, niacinamide, doxycycline, tetracycline, antibiotics, and dapsone). This study used systematic glucocorticoid (95.12%) and topical steroids (73.17%) to treat most cases. Our findings showed that pembrolizumab-induced BP required several months of systemic maintenance therapy with corticosteroids, in contrast to dipeptidyl peptidase-4 inhibitors-induced BP, which typically resolves by discontinuing the drug (82). However, routine glucocorticoids application not only causes more adverse reactions but may also lessen the effectiveness of immunotherapy (83). Other immunosuppressants recommended for classical BP should be used with caution in pembrolizumab-induced BP because of the possibility of accelerating the progression of tumors (76). Therefore, treatment choice depends on the patient’s condition, especially the severity of BP, pre-existing cancer, and the presence of comorbidities.

The use of biological therapies, such as omalizumab, dupilumab, and rituximab, is a potential alternative for treating severe, relapsing or refractory cases. A systematic review of 211 patients included 122 patients receiving rituximab, 53 patients receiving omalizumab, and 36 patients receiving dupilumab, with the majority of patients receiving corticosteroids, immunosuppressants, and antibiotics but failing to be treated before biologic drug treatment (84). In this study, rituximab, omalizumab, and dupilumab led to complete remission in 70.5%, 67.9%, and 66.7% of patients and partial remission in 23.8%, 20.8%, and 19.4% of patients. Additionally, most of these patients who received the biologicals had no side effects. This suggested that rituximab, omalizumab, and dupilumab could be alternatives to traditional therapy for refractory BP. However, the exact mechanism through which biologicals (i.e., omalizumab, dupilumab, and rituximab) result in clinical remission in BP is still unclear. The key potential mechanisms were that omalizumab blocked IgE from binding to cell surface FcϵRI, dupilumab blocked the signaling pathway of IL-4 and IL-13, and rituximab hindered the surface protein CD20 expressed on B-cell lymphocytes, leading to BP remission (84). Additionally, our findings also demonstrated that using rituximab or dupilumab resulted in complete remission of BP in 4 patients with poor response to systemic glucocorticoids combined with nonbiologic agents. Furthermore, one patient in our results who failed glucocorticoid therapy did not experience remission after infliximab therapy. This finding suggests that dupilumab or rituximab may be effectively control the disease in patients with pembrolizumab-induced BP. The earlier incorporation of biologics (rituximab, dupilumab, omalizumab) into the treatment regimen merits consideration in refractory or complicated cases with pembrolizumab-induced BP. However, additional research is required to clarify the efficacy and safety of biological targeted agents like omalizumab, rituximab, dupilumab, and infliximab in this patient population. Although the treatments of biologics (rituximab, dupilumab, and omalizumab) are off-label in BP and there is no strong evidence supporting their use as a first-line treatment, research into molecular target therapies for BP is currently underway worldwide, especially in the United States (85). The clinical practice and standard of care for ICIs-induced BP at major medical centers in developed countries, such as the United States, have turned or are turning to dupilumab or rituximab as first-line treatments to minimize the need for ongoing immunosuppression and the negative consequences of systemic corticosteroid therapy. However, due to the high cost of dupilumab or rituximab, glucocorticoids are still used as first-line therapy for ICIs-induced BP in less developed countries such as China.

This study has some limitations. First, it was different from the evidence gathered for many years for classical BP. This study used the published case reports/series, not a cohort of patients, for secondary analysis. However, the case reports and case series included in the analysis of this study lack consistency in the data and in the level of detail reported. Therefore, publication bias was a significant limitation of our study. Second, because BP is a classic disease, selective reporting in the literature may be possible, with fatal cases more likely to be reported than non-fatal ones. This could result in an underestimation. Third, because our study only looked at pembrolizumab-induced BP and not BP induced by all immune checkpoint inhibitors, we could not determine BP caused by different immune checkpoint inhibitors. Additional research is required to assess the differences in BP caused by different immune checkpoint inhibitors. Moreover, we were not able to describe the risk factors and severity of BP caused by pembrolizumab because they were rarely reported in case reports/series, which prevented further analysis. Finally, we included a small number of cases and lacked of a control group in the analysis. Larger, cohort studies are necessary to establish the clinical characteristics, diagnosis, management and prognosis of pembrolizumab-induced BP. Furthermore, it is crucial to develop definitive, cost-effective, and reproducible biomarkers biomarkers to optimize anti-tumor regimens in patients with cancer who are at a higher risk of developing BP.

## 5 Conclusions

This study on pembrolizumab-induced BP illustrates the spectrum of clinical manifestations, serological, histopathological, and immunopathological characteristics and effects on tumor treatment. Clinicians should be mindful BP when patients receive pembrolizumab or complete its therapy and show develop prodromal pruritus and other nonspecific cutaneous symptoms. It is possible to confirm the diagnosis of BP with the help of a skin biopsy, immunologic tests, and serum tests, particularly for anti-BP180 autoantibody. Most patients with pembrolizumab-induced BP had clinical and histopathological findings consistent with the classic BP. Patients may need to terminate pembrolizumab and maintain systemic corticosteroids for several months while receiving nonbiologic medications (i.e., cyclophosphamide, methotrexate, azathioprine, mycophenolate mofetil, immune globulin intravenous, niacinamide, doxycycline, tetracycline, antibiotics, and dapsone). Further studies are needed to explain the underlying mechanisms of pembrolizumab-induced BP, identify patients at risk of developing BP, and determine the most effective therapeutic interventions and preventative measures regarding their ability to control symptoms without compromising the anti-tumor efficacy of immunotherapy.

## Data availability statement

The original contributions presented in the study are included in the article/supplementary material. Further inquiries can be directed to the corresponding author.

## Ethics statement

Ethical review and approval was not required for the study on human participants in accordance with the local legislation and institutional requirements. Written informed consent for participation was not required for this study in accordance with the national legislation and the institutional requirements.

## Author contributions

WL and CW designed the study. JW, XH, WJ, WZ, MT, and XZ prepared the manuscript. All authors contributed to the article and approved the submitted version.
